# Predicting youth intention to help a road accident victim in fast urbanizing district of India: a PLS-SEM approach based on the Theory of Planned Behavior

**DOI:** 10.5249/jivr.v15i1.1770

**Published:** 2023-01

**Authors:** Neeti Rustagi, Abhishek Jaiswal, Naveen Dutt, Arvind Sinha, Pankaja Raghav, Vikas Rajpurohit

**Affiliations:** ^*a*^ Department of Community & Family Medicine, All India Institute of Medical Sciences, Jodhpur, Rajasthan, India.; ^*b*^ Senior Resident, Centre for Community Medicine, All India Institute of Medical Sciences, New Delhi, India.; ^*c*^ Department of Pulmonary Medicine, All India Institute of Medical Sciences, Jodhpur, Rajasthan, India.; ^*d*^ Department of Pediatric Surgery, All India Institute of Medical Sciences, Jodhpur, Rajasthan, India.; ^*e*^ Hospital & Dr. S. N. Medical College Jodhpur, Rajasthan, India.

**Keywords:** Accidents, Traffic, Emergency Responders, Factor Analysis, Statistical, Intention, Psychometrics

## Abstract

**Background:**

In countries with evolving prehospital trauma care systems, it is recommended that volunteers es-pecially youth can be trained to perform as first responders to render basic emergency care until care by formally trained health-care personnel’s is available. Based on the theory of planned behavior (TBP), the present study aims to predict intention to help road accident victim among young adults in a fast-urbanizing Indian city.

**Methods:**

A cross sectional survey was conducted among 695 college students of Jodhpur, Rajasthan by self-administered questionnaire based on theory of planned behavior (TPB). Predictor of behavioral intention to help an accident victim was assessed through partial least square structural equation model (PLS-SEM).

**Results:**

Theory of planned behavior provided a reliable and valid framework for predicting intention of college students towards helping an accident victim. Perceived confidence (β = 0.344, p less than 0.001); attitude (β = 0.323, p less than 0.001) and social norm (β = 0.251, p less than 0.001), all emerged as the significant direct predictor of intention. Perceived confi-dence also significantly predicted social norm (β = 0.370, p less than 0.001) and attitude (β = 0.281, p less than 0.001). Further, attitude towards helping an accident victim was also influenced by social norm (β = 0.366, p less than 0.001).

**Conclusions:**

Based on framework of TPB, role of perceived confidence, social norm and attitude is found to significantly predict intention among college youth towards helping an accident victim. Public health interventions designed towards engaging and training youth as first responders in countries with fragmented pre-hospital trauma care systems need to encompass these aspects by establish-ing community based training programs for potential first responders and recognition of good Samaritans.

## Introduction

Road safety is priority area for research as studies from the World Health Organization (WHO), (2018), reports 1.35 million death and 50 million injuries on the world’s roads every year.^1^ In developing countries including India , risk of dying in road traffic crash is much higher than in high-income countries due to delayed post-crash care and long distance from trauma centres.^2,3^ Thus, strengthening the community-based systems that serve as the first point of contact is essential to ensure timely and equitable access to all accident victims. 

As per Sasser et al.^4^ substantial reduction of deaths of severely injured RTA patients is possible by early administration of basic ﬁrst aid measures through trained volunteers and non-health professionals. Given the gaps in organized prehospital care in many low- or middle- income countries, bystanders and laypeople are recommended to be taught the basics of care to trauma victims.^5^

Bystanders are people who attend at the crash scene without any knowledge and skill about relief, rescue, and first aid to the RTAs victims.^6^ However, in some cases, bystanders with adequate understanding about aiding the injured can perform basic life support measures before arrival of the emergency organizations or may provide transportation.^7,8^

Prosocial behaviour or helping behaviour was defined by Stang et al.^9^ as a voluntary behaviour performed with the intention of benefiting another person or group of people. Situational characteristics in form of “diffusion of responsibility”; “ambiguity in interpreting situation” and “cost reward analysis of the helping act” along with individual personality traits such as “adherence to norm of social responsibility”; “empathy”; “pro-social values”; “level of moral development” are hypothesized predictors determining helping behaviour especially in emergency situation.^10-13^

When teaching a new skill such as first response, educators focus mostly on the what and how (cognitive and psychomotor skills) rather than the why, which falls under the affective domain of learning.^14^ Previously several studies have reported that information about helping or acquisition of skills does not always increase bystander responsiveness.^8,15,16^

The approach regarding bystander training in form of self-directed learning or instructor-led course focuses upon building individual skills acquisition and retention. However, evaluation of a learner’s intention of volunteering help at incident site or theory-based attempts to strengthen such behaviours are rarely reported. Further, poor understanding exists regarding predictors likely to influence young adults’ intention for providing post-crash care. 

According to The Theory of Planned Behaviour (TBP) model, an individual’s behavioural intentions influence their motivation for engaging in a particular behaviour.^17^ The TBP is a social cognitive model and predicts that an individual’s action largely reflects their attitudes, perceived norms and accepted behaviours and how well they believe they can perform the task.^18,19^ Intention focused model to enhance performance of CPR by bystanders was conceptualized by Panchal et al.^20^ Given the motivational component in providing post-crash care, TPB may provide a useful framework to investigate the young adult intention at site of road traffic accident. This study aimed to predict the youth intention towards helping a road accident victim based on social norms, attitude and perceived confidence in fast urbanizing city of Jodhpur, Rajasthan.

**Following hypothesis were tested**

H1: Higher perceived behavioural control toward helping accident victims would significantly predict bystanders’ intention to help road traffic accident victims

H2: Positive attitude toward helping accident victims would significantly predict bystanders’ intention to help road traffic accident victims. 

H3: Positive social norm toward helping accident victims would significantly predict bystanders’ intention to help road traffic accident victims.

H4: Higher perceived behavioural control toward helping accident victims would significantly predict bystanders’ attitude to help road traffic accident victims.

H5: Higher perceived behavioural control toward helping accident victims would significantly influence bystander’s normative beliefs (social norms) to help road traffic accident victims.

H6: Positive social norm toward helping accident victims would significantly predict bystanders’ attitude to help road traffic accident victims.

## Methods 

This study was conducted as part of a funded project carried out to evaluate pre-hospital trauma care systems in Jodhpur, Rajasthan. This study consisted of two phases: Phase 1- questionnaire development and expert panel review and Phase 2- validation process.

**Phase 1: Questionnaire development**

Literature search was conducted regarding predictors related to bystander’s behavioural intention to help road accident victims. Combination of keywords included “helping behaviour”, “accidents”, “traffic accidents”, “bystanders”, “theory of planned behaviour” was used on search engines as PubMed, Science Direct and Google Scholar. Instrument to measure young adults’ behavioural intention was framed and scored as per the constructs of theory of planned behaviour.^21,22^

The questionnaire contained questions as per TPB constructs regarding participants behavioural intention, attitude, subjective norm and perceived behavioural control towards helping road accident victim.^23,24^ Each item was measured on a 7-point Likert scale where higher scores reflected desired or expected response. Attitude was assessed based on behavioural beliefs related to consequences regarding behaviour and was assessed through items such as “Providing appropriate care to an accident victim will likely save life of patient”. Beliefs about perceived opinion of significant others determine social norms and was determined by asking feedback related to friends and work place colleagues. Perceived behavioural control was assessed based on perceived ease or difficulty about performing the behaviour required to help accident victim. For assessing intention, items assessing situational confidence and intention towards helping a road side accident victim at accident site and establishing necessary communication with other bystander’s and health care teams were included. 

The original questionnaire items in English language were translated into local Hindi language to suit the needs of study participants. Help of linguistic expert and a medical doctor was sought for two forward translations of original English questionnaire into Hindi followed by backward translation into English by another linguistic expert and a medical doctor. This was to ensure Hindi translation of items to match the original English version. 

**Phase 2- Validation process**

Face and content validity of questionnaire was assessed. For content validity, experts in field of emergency and trauma did peer review and edits were made based on provided feedback. For face validity, ten young adults as considered sufficient for pilot testing^25^ were cognitively debriefed before administering questionnaire. The respondents were interviewed and asked to identify words or sentences that they did not understand and were encouraged to suggest alternate suitable familiar words. The feedback of participants was discussed with other investigators in the research team while preparing final version of questionnaire. The final version of questionnaire used in the study is given in Appendix  as survey instrument.

**Appendix:** Survey Instrument

Name	City which you originally belong
Semester	Age
Course	Sex
A1 Experience of helping a road side accident victim at accident site till transfer to health facility is		Unpleasant: ___1__:___2__:___3__:___4__:___5__:___6__:___7___: pleasant	
SN_1 My friends will appreciate me helping a roadside accident victim		Unlikely: ___1__:___2__:___3__:___4__:___5__:___6__:___7___: likely	
I1 I am confident that I can help a road side accident victim at accident site till transfer to health facility		Disagree: ___1__:___2__:___3__:___4__:___5__:___6__:___7___: agree	
I2 I am confident in establishing necessary communication with health care team while taking care of an accident victim		Disagree: ___1__:___2__:___3__:___4__:___5__:___6__:___7___: agree	
I3 I intend to help a road side accident victim till they transfer to health facility		Unlikely: ___1__:___2__:___3__:___4__:___5__:___6__:___7___: likely	
P1 I feel confident in my ability to manage road side accident victim.		Disagree : ___1__:___2__:___3__:___4__:___5__:___6__:___7___: agree	
I4 I am confident in establishing necessary communication with bystanders at accident site		Disagree: ___1__:___2__:___3__:___4__:___5__:___6__:___7___: agree	
A2 Providing appropriate pre hospital trauma care to an accident victim will likely save life of patient		Disagree: ___1__:___2__:___3__:___4__:___5__:___6__:___7___: agree	
A3 Helping a road side accident victim till they reach health facility is my preferred choice		Disagree: ___1__:___2__:___3__:___4__:___5__:___6__:___7___: agree	
P2 I am confident in assessing and managing breathing in trauma patient		Disagree: ___1__:___2__:___3__:___4__:___5__:___6__:___7___: agree	
SN_3 People from my neighborhood are likely to help a road side accident victim at accident site till transfer to health facility		Unlikely: ___1__:___2__:___3__:___4__:___5__:___6__:___7___: likely	
A4 Giving care to a roadside accident victim before referring to health facility is life saving skill		Disagree: ___1__:___2__:___3__:___4__:___5__:___6__:___7___: agree	
P3 I am confident in assessing and managing hemorrhage in trauma patient		Disagree: ___1__:___2__:___3__:___4__:___5__:___6__:___7___: agree	
P4 I am confident in assessing and managing in spinal care to trauma patient		Disagree: ___1__:___2__:___3__:___4__:___5__:___6__:___7___: agree	
SN_2 My work place colleagues will appreciate me Helping a road side accident victim		Unlikely: ___1__:___2__:___3__:___4__:___5__:___6__:___7___: likely	

The final questionnaire was administered among students of academic institutions of urban Jodhpur, Rajasthan from July 2019 till October 2019. Data of educational institutions was availed through annual report of year 2018 -2019 from Government of Rajasthan website for higher and technical education.^26^ The colleges of medical and other allied disciplines (pharmacy, nursing, and dental) were excluded, as these students are not representative of general population due to their chosen study of discipline. Out of identified twenty-four professional colleges, six were randomly chosen by lottery method (without replacement) whose students were likely to possess negligible or limited training to act at RTA site. Learning opportunities for non- health discipline students to learn initial trauma care are rare and are not part of academic curriculum in this region. All the students present in these institutes were approached for the study purpose through the college administration and were repeatedly reminded to participate. A print version of the questionnaire was administered to the students in 30 minutes’ class room setting on pre-specified days. Two research assistants were present throughout to support students. 

**Ethics approval: **This study was part of an Indian Council of Medical Research project approved by the AIIMS Jodhpur Ethics Committee. Ethical clearance for performing this study was obtained from the Institutional Ethics Committee (AIIMS / IEC/2018 /1188, dated 02.05.2018). All the eligible participants were informed about the purpose of the study, and were assured regarding the confidentiality of the information obtained. Written informed consent was obtained and no monetary reward was provided for willing participants. The items were not compulsory and participants had the option of abstaining from each question. The confidentiality of the dataset was maintained by removing the identifiers. All procedures performed in studies involving human participants were in accordance with the ethical standards of the institutional and/or national research committee and with the 1964 Helsinki declaration and its later amendments or comparable ethical standards.

**Statistical analysis: **Responses were coded, and entered in Microsoft excel. No items were reverse coded for data analyses. The distribution of the responses was inspected for each item to eliminate items with a low discriminative power. These were (a) items with 95% or more of the given answers in the same category, and (b) items with a standard deviation lower than .75. 

The statistical analyses were performed using the SPSS Version 25.0 software package for Windows (SPSS, Inc., Chicago, IL, USA) for descriptive analysis. SmartPLS4.0. was used to build reflective measurement model and test the proposed hypothesis using partial least squares- structural equation modelling (PLS-SEM). PLS-SEM is preferred when the collected data is non-normal ; considered suitable for exploratory study which involves developing new ideas and research objective is based on prediction.^27^

For measurement model, the reliability of measures was evaluated both on indicator and construct level. For validity assessment, convergent and discriminant validity was assessed.

Indicator reliability: In reflective measurement model, the indicator reliability is established by amount of each indicator variance explained by its construct which is calculated by squaring of indicator loading. Loadings greater than 0.708 is considered acceptable as it establishes more than 50% of variance being explained by construct.^28^ Internal consistency reliability: This reflects the extent to which indicators for a construct are associated with each other and is demonstrated by Cronbach’s alpha, composite reliability and reliability coefficient.^28^ Values between 0.70 to 0.90 are considered satisfactory to good while between 0.60 to 0.70 are considered to be acceptable.^28^

Convergent validity is assessed based on average variance extracted (AVE) of construct and value more than 0.5 is considered acceptable and establishes that construct explains at least 50% of indicator variance.^30^ The constructs' discriminant validity is assessed as per heterotrait – monotrait method of correlations. In view of conceptually distinct constructs, threshold value of 0.85 was considered for this study.^30^

For structural model,(1) the coefficients of determination of the endogenous variables (R2 values) and the predictive relevance was determined by Stone–Geisser Q2 values where values of 0.02, 0.15 and 0.35 represent small, medium and large effects.^27^

Thereafter, the quantification of the hypothesized relationships within the inner model was done as the main analysis. Path coefficients and the effect sizes were assessed. For effect size ( f 2) values of 0.02, 0.15 and 0.35 represent small, medium and large effects, respectively.^27^

## Results

Of 695 participants who responded, after removing the data with missed responses for one or more questions 662 were included. 

Sample characteristics: Mean age of male participants (mean 19.18 years; SD 1.9) was significantly higher compared to female (mean 18.82; SD 1.8) participants [ t (660) = 2.49; p=0.013]. No significant difference was observed in formal years of education of male and female participants. Significantly more male participants (37.2%) as compared to female participants (23.1%) had previous experience of helping a road traffic accident victim [ chi square (1) = 15.30; p <0.001] 

Item analysis: Mean and standard deviation of behavioural domain and included items is illustrated in Table 1 Although some of the items were skewed, all showed sufficient variation across the response categories (i.e., less than 95% of responses on a single category and SD more than 0.75). 

**Table 1:** Mean score of items (n=662).

Construct	Item	Mean	SD
Attitude	A1	3.83	2.135
	A2	5.59	1.712
	A3	5.85	1.450
	A4	5.63	1.585
Social Norm	SN_1	5.42	1.889
	SN_2	5.27	1.743
	SN_3	4.96	1.707
Perceived confidence	P1	5.08	1.639
	P2	4.33	1.898
	P3	4.35	1.908
Intention	I1	5.43	1.649
	I2	5.22	1.671
	I3	5.63	1.605
	I4	4.96	1.791

Measurement model: Construct reliability and validity for the measurement model was assessed. For indicator reliability, the indicator loadings less than 0.6 were deleted if it leads to increase in the respective constructs’ composite reliability. Internal consistency reliability was sufficient as value for Cronbach’s alpha, composite reliability and reliability coefficient was in range of 0.6 to 0.9. Convergent validity was established as average variance extracted was more than minimum threshold value of 0.5 for all constructs. (Table 2) 

**Table 2:** Factor loadings, reliability and convergent validity of measurement model.

Item←Constructs	Outer loadings	Cronbach's alpha	Reliability coeffi-cient (rho A)	Composite reliability	Average variance extracted
Item SN_1←Social Norm	0.878	0.608	0.623	0.835	0.717
Item SN_2←Social Norm	0.814				
Item I1←Intention	0.792	0.770	0.773	0.853	0.592
Item I2←Intention	0.764				
Item I3←Intention	0.789				
Item I4←Intention	0.731				
Item A2←Attitude	0.826	0.623	0.634	0.841	0.725
Item A3←Attitude	0.877				
Item P1←Perceived confidence	0.735	0.673	0.696	0.817	0.598
Item P2←Perceived confidence	0.771				
Item P3←Perceived confidence	0.811				

Discriminant validity was achieved as the heterotrait- monotrait ratio was less than 0.85 for all constructs.(Table 3)

**Table 3:** Discriminant validity of measurement model by Heterotrait-Monotrait Ratio.

	Attitude	Intention	Perceived confidence
**Attitude**			
**Intention**	0.833		
**Perceived confidence**	0.644	0.797	
**Social Norm**	0.76	0.769	0.546

**Figure 1:**
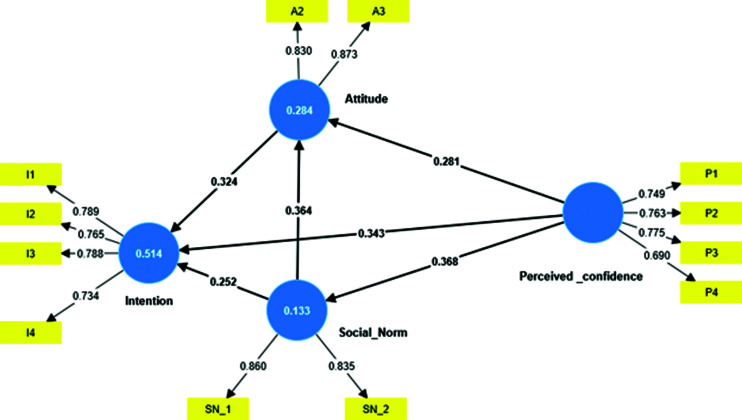
Summary of structural model with adjusted R2; path coefficients and factor loadings.

Structural model: Based on theory of planned behaviour, structural model was created. (Figure 1) Collinearity test of inner model was evaluated through variance inflation factor (VIF) which was below 2.0 for each of the constructs, as suggested by Hair et al. (2014).^31^ This was followed by assessing the co-efficient of determination (R^2^) and the effect size (f^2^) on the impact value of the perceived confidence on other constructs. The predictive accuracy of the model predictions (Q^2^) was also assessed. The Q^2^ of the full structural model was above zero, and the (unreported) VIF was less than 2 for all predictor constructs. The exogenous variables in the model were able to explain 51.9% of variance in intention to help. The R^2^ value of the attitude was 0.170 while for social norm was 0.135 suggesting that perceived confidence explained 17% and 13.5% of attitude and social norm construct respectively. 

Attitude (f^2^ = 0.155) and perceived confidence (f^2^ = 0.193) has medium effect size on behavioural intention as compared to social norms (f^2^ = 0.098) where effect size was small. The effect size of perceived confidence on social norms (f^2^ = 0.156) and social norms on attitude (f^2^ = 0.161) was medium. This was as per Cohen recommendations.^32^

The Q2 values for the attitude (0.165), intention (0.318), and social norm (0.129) were greater than zero which confirmed model’s predictive relevance to be adequate for the exogenous variable perceived confidence.^33^

**Relationship among the constructs**

Six hypotheses (H1 to H6) postulated the direct association between the predictors and intention. Table 4 illustrates the bootstrapping results based on sub-samples of 5,000 cases to examine the relationship among the constructs. All the hypotheses were analysed simultaneously.

**Table 4:** Summary of structural model.

	Hypothesized path	Path coefficient	Standard error	T values	P values	Decision
H1	Perceived confidence→Intention	0.344	0.037	9.356	0.000	Supported
H2	Social norm→Intention	0.251	0.044	5.781	0.000	Supported
H3	Attitude→Intention	0.323	0.041	7.915	0.000	Supported
H4	Perceived confidence→attitude	0.281	0.042	6.710	0.000	Supported
H5	Perceived confidence→Social norm	0.370	0.043	8.546	0.000	Supported
H6	Social norm→attitude	0.366	0.046	7.856	0.000	Supported

Social norm (R^2^ - 0.135; f^2^ - 0.129; Q^2^- 0.129) Attitude (R^2^ - 0.287; f^2^ - 0.165; Q^2^- 0.165) Intention (R^2^ - 0.517; f^2^- 0.318; ; Q^2^- 0.318)

Perceived confidence (β = 0.344, p<0.001); attitude (β = 0.323, p<0.001) and social norm (β = 0.251, p<0.001), all emerged as the significant direct predictor of intention. Perceived confidence also significantly predicted social norm (β = 0.370, p<0.001) and attitude (β = 0.281, p<0.001). Further, attitude towards helping an accident victim was also influenced by social norm (β = 0.366, p<0.001). 

## Discussion

This study established that behavioural intention to help an accident victim among educated youth can be well explained and predicted by psycho-social factors based on TPB theory i.e. attitude, social norms and perceived confidence. Ability of model to predict more than half of variance in behavioural intention is appreciable as the behavioural learning takes into account knowledge, skills and affective domain.^34^

PLS- SEM yielded four factors aligning with the TPB constructs. Convergent and discriminant validity of scale was established. Standardized internal consistency of scale was satisfactory (Cronbach’s alpha and omega coefficient >0.6) for all constructs. 

This study thus establishes that the developed tool is valid and reliable to evaluate behavioural intention of young adults for providing assistance at RTA site. 

**Implications for Prevention and Intervention Strategies:**

Considering the perceived confidence emerged as one of the significant predictor influencing behavioural intention, attitude and social norms, countries with high burden of accidents and developing trauma care systems should consider training community volunteers as first responders.^5^ Assessing behavioural intention among different subgroups of population reportedly helps in understanding gaps while engaging learners in training activities.^35^

Bhalla et al.^36^ reported the challenges faced by good Samaritans specifically by Indian medico-legal systems while helping an accident victim. In current study, social norms were found to significantly influence both behavioural intention and attitude towards helping an accident victim. Thus, measures facilitating role of good Samaritans and their social recognition need to be considered for strengthening bystanders’ intervention towards accident victim.^37^

Considering the lifesaving nature of pre-hospital trauma care education, it is recommended that countries with evolving trauma care systems should train community volunteers as first responders to facilitate early and accurate care. ^5^ Attention to behavioral intention and measuring it within population subgroups is essential to identify and fill gap in understanding of the learner and to effectively engage them in such community based interventions.^35^

Further, our study provides initial evidence highlighting need of integrating components addressing attitude, social norms and perceived confidence of participants (cognitive, psychomotor and affective domain) based on theory of planned behavior for trauma educators working towards strengthening community based early response mechanism for accidents and disasters. 

Few of the major limitations in this study is the self-reported data by participants which might not represent actual behaviour of participants.^38^ Also, no formal definition or exact nature of helping behaviour was outlined and thus participants understanding about engaging in helping behaviour at accident site might not be identical. 

Future research is required to evaluate the generalizability of current findings among young adults out of formal education and from different population subgroups in form of ethnicity, age, gender and socio-economic background. Further studies are needed to evaluate participants behavioural intention towards RTA victim in pre-post learning environment. Presence of an intent-to-act may not necessarily result in actual act^39^ thus, longitudinal studies assessing predictors of helping behaviour at the site of road accident or conducting experimental deception-based studies to identify actual behaviours will be helpful. 

**Conclusion: **This study established the validity and reliability of theory of planned behaviour in predicting behavioural beliefs of young adults towards helping an accident victim. Public health advocacy, community-based training programs and social recognition of good Samaritans appear as an effective strategy towards strengthening post-crash care in places with evolving trauma care systems. 

**Abbreviations:**

AVE: Average variance extracted

PLS-SEM: Partial least square structural equation model

RTA: Road traffic accidents

SD: Standard Deviation

TBP: Theory of Planned Behaviour 

VIF: Variance inflation factor

WHO: World Health Organization

**Declarations:**

**Ethics approval and consent to participate: **The study was approved by All India Institute of Medical Sciences, Jodhpur Ethics Review committee (number: AIIMS/IEC/2018/488) and was performed in accordance with the ethical standards ascribed by the 1964 Declaration of Helsinki and its later amendments. Approval was further obtained from the authorities of selected colleges. This study only involved participants who provided their written informed consent. The questionnaire was treated as confidential and anonymous; there was no personal information that could link the responses with any of the participants in the study. Each completed questionnaire was returned to the researcher on the same day of data collection.

**Acknowledgments**

We are grateful for input from our clinical partners, including those who reviewed study documents and provided suggestions. We are also grateful to participants for sharing their perspective with us. We wish to thank the dean/ principals of academic institutions for providing us necessary permissions and support to conduct the study.
